# Efficacy and prognostic factors of COVID‐19 vaccine in patients with hepatocellular carcinoma: Analysis of data from a prospective cohort study

**DOI:** 10.1002/cam4.70068

**Published:** 2024-08-09

**Authors:** He Zhao, Ying Li, Pengfei Tian, Wei Sun, Yingen Luo, Xiaowu Zhang, Jingui Li, Tao Gong, Zhengqiang Yang, Peng Song, Xiao Li

**Affiliations:** ^1^ Department of Interventional Therapy, National Cancer Center/National Clinical Research Center for Cancer/Cancer Hospital Chinese Academy of Medical Sciences and Peking Union Medical College Beijing China; ^2^ Department of Interventional Therapy, National Cancer Center / National Clinical Research Center for Cancer / Cancer Hospital and Shenzhen Hospital Chinese Academy of Medical Sciences and Peking Union Medical College Shenzhen China; ^3^ Department of Interventional Therapy First Hospital of China Medical University Shenyang China

**Keywords:** clinical observations, hepatocellular carcinoma, prognosis, vaccine

## Abstract

**Background:**

The efficacy of coronavirus disease 2019 (COVID‐19) vaccines in preventing SARS‐CoV‐2 infection in patients with hepatocellular carcinoma (HCC) is not clear.

**Methods:**

From January 2022 to October 2022, patients diagnosed with HCC in a prospective, multicenter, observational cohort were analyzed.

**Results:**

One hundred and forty‐one patients with (*n* = 107) or without COVID‐19 vaccination (*n* = 34) were included. The number of patients with severe or very severe infection was relatively lower in the vaccinated group (3.7% vs. 11.8%, *p* = 0.096). Median infection‐free survival in the vaccinated group (14.0 vs. 8.3 months, *p* = 0.010) was significantly longer than that in the unvaccinated group. COVID‐19 vaccination (hazard ratio (HR) HR = 0.47), European Cooperative Oncology Group performance score = 0 (HR = 2.06), and extrahepatic spread (HR = 0.28) were found to be the independent predictive factors for infection‐free survival.

**Conclusion:**

COVID‐19 vaccines could effectively reduce the SARS‐Cov‐2 infection in patients with HCC.

## 1 INTRODUCTION

Hepatocellular carcinoma (HCC) represents approximately 85% of primary liver cancer, and it is unique because it usually occurs in patients with underlying chronic liver diseases, which may cause a number of abnormalities, including abnormal blood tests, impaired immunological reactions, or even death from complications of chronic liver diseases.^1^, ^2^ In comparison to healthy adults, patients with HCC were noted to have higher mortality after the SARS‐CoV‐2 infection.^3^ Moreover, in patients with chronic liver disease, HCC was shown to be an independent risk factor for higher mortality associated with SARS‐CoV‐2 infection.^4^ In studies of patients with HCC, the safety of the coronavirus disease 2019 (COVID‐19) vaccine was found to be acceptable. However, the immunogenicity of the COVID‐19 vaccine was found to be lower than that of other patients or healthy adults.^5^, ^6^ Given the paucity of definitive evidence, the efficacy of administering COVID‐19 vaccines to this population remains a topic of debate. This study aims to investigate the efficacy and prognostic factors of COVID‐19 vaccines for preventing SARS‐CoV‐2 infection in patients with HCC, to inform future strategies for the prevention and management of global infectious diseases.

## 2 MATERIALS AND METHODS

### 2.1 Study design

The prospective, multicenter, observational Primary Liver CanCer cohort (PLCC cohort), which was approved by the ethics committee of Cancer hospital, Chinese Academy of Medical Sciences, and registered in Chinese Clinical Trial Registry (identifier: ChiCTR2200056326), enrolled all consecutive patients, who were diagnosed with primary liver cancer, had no history of local or systemic treatments and voluntarily consented to participate in the cohort since January 2022. The PLCC cohort aims to prospectively observe and record the general characteristics (e.g., vocation, social support, laboratory parameters, radiological and pathological features) and the health behaviors (e.g., nutritional status, exercise, COVID‐19 vaccination status) in patients with primary liver cancer, and further investigate the relationship between the factors and their prognosis. Informed consents were collected from all patients. From January 2022 to October 2022, all patients (≥18 years old) diagnosed with HCC in the PLCC cohort were considered to be eligible for the analyses. Patients with other pathological types of primary liver cancer were excluded. To evaluate the efficacy of vaccines in preventing SARS‐CoV‐2 infection, patients who had a history of SARS‐CoV‐2 infection before enrollment were excluded. To investigate the relationship between the COVID‐19 vaccination and overall survival, a certain period of follow‐up (e.g., 12 months) was necessary. In addition, the most significant outbreak of COVID‐19 in mainland China occurred at the end of 2022.^7^ Prior to this, the infection rate was merely 0.7‰, as a result of the dynamic zero‐COVID policy in mainland China.^8^ Therefore, October 2022 was decided as the deadline for enrollment in this study.

### 2.2 Data collection and follow‐up

Baseline clinical, laboratory, radiological, and COVID‐19‐related data (e.g., vaccination status, type of vaccine, vaccine‐related adverse reactions) were prospectively collected immediately after enrollment. Subsequent follow‐up visits were scheduled at 6‐week intervals to collect information regarding SARS‐CoV‐2 infection, adverse reactions, survival, and other pertinent data. Vaccine‐related adverse reaction was defined as adverse reactions occurring within seven days of the injection, which were related to the COVID‐19 vaccination.^9^ The relationship between the adverse reactions and vaccination was determined by the investigators. Infection‐free survival was defined as the time from enrollment to the SARS‐CoV‐2 infection. Overall survival was defined as the time from enrollment to death from any cause.

## 3 RESULTS

### 3.1 Patient recruitment

A total of 165 patients were screened during the study period. None of the patients had a history of SARS‐CoV‐2 infection. Patients with intrahepatic cholangiocarcinoma (*n* = 10), combined hepatocellular‐cholangiocarcinoma (*n* = 7), hepatic sarcoma (*n* = 2), neuroendocrine neoplasm (*n* = 2), sarcomatoid carcinoma (*n* = 2), and cancer of unknown primary origin (*n* = 1) were excluded. No patient had a history of SARS‐CoV‐2 infection before enrollment. Finally, a total of 141 patients with HCC (85.5%) were included in the analyses (Figure S1). One hundred and seven patients (75.9%) who received at least one dose of the COVID‐19 vaccine were categorized into the vaccinated group, and the other 34 (24.1%) patients were categorized into the unvaccinated group.

### 3.2 Baseline characteristics

Age, sex, etiology, Child‐Pugh class, European Cooperative Oncology Group (ECOG) performance score, maximum tumor diameter, number of lesions, portal invasion, extrahepatic spread, serum albumin, total bilirubin, prothrombin time, creatinine, alpha‐fetoprotein, comorbidities, and some socioeconomic factors were similar between the two groups (Table S1). In the vaccinated group, patients classified as BCLC (Barcelona Clinic Liver Cancer Staging system) stage B were significantly higher than in the unvaccinated group (30.8% vs. 11.8%, *p* = 0.044). The median follow‐up was 16.3 months (interquartile range: 14.2–19.3 months). Seven (5.0%) patients (vaccinated vs. unvaccinated = 5 vs. 2) were lost to follow‐up after a median follow‐up of 9.7 months (interquartile range: 1.8–11.1 months). Nineteen patients received immunotherapy (vaccinated vs. unvaccinated = 16 vs. 3, *p* = 0.533) during the follow‐up period.

### 3.3 Characteristics of the COVID‐19 vaccination

A total of 259 doses of the COVID‐19 vaccines were administered to the vaccinated group (Table S1). One hundred and seven patients (75.9%) received at least one dose of the COVID‐19 vaccine. Among these patients, 93.5% received the standard vaccination (i.e., two doses of vaccine), and 48.6% received the booster vaccination. The two most commonly administered vaccines were CoronaVac (Sinovac Life Sciences, China) (48.6%) and BBIBP‐CorV (Beijing Institute of Biological Products, China) (37.4%). A total of 37 vaccine‐related adverse reactions occurred in 24 patients (24/107, 22.4%) after 259 doses of COVID‐19 vaccines. Furthermore, the vaccine‐related adverse reactions occurred in 15.8% (17/107), 11.0% (11/100), and 17.3% (9/52) of the patients following the first, second, and third doses of the COVID‐19 vaccine, respectively (Table S2). One patient with a history of psoriasis exhibited a significant exacerbation of psoriasis symptoms following each of the two doses of the COVID‐19 vaccine. The remaining vaccine‐related adverse events were mild or moderate in severity, including local pain at the injection site (8.5%), fatigue (2.3%), vertigo (1.5%), cough (1.2%), sleepiness (1.5%), and fever (0.8%).

### 3.4 SARS‐CoV‐2 infection and COVID‐19 vaccination

**FIGURE 1:**
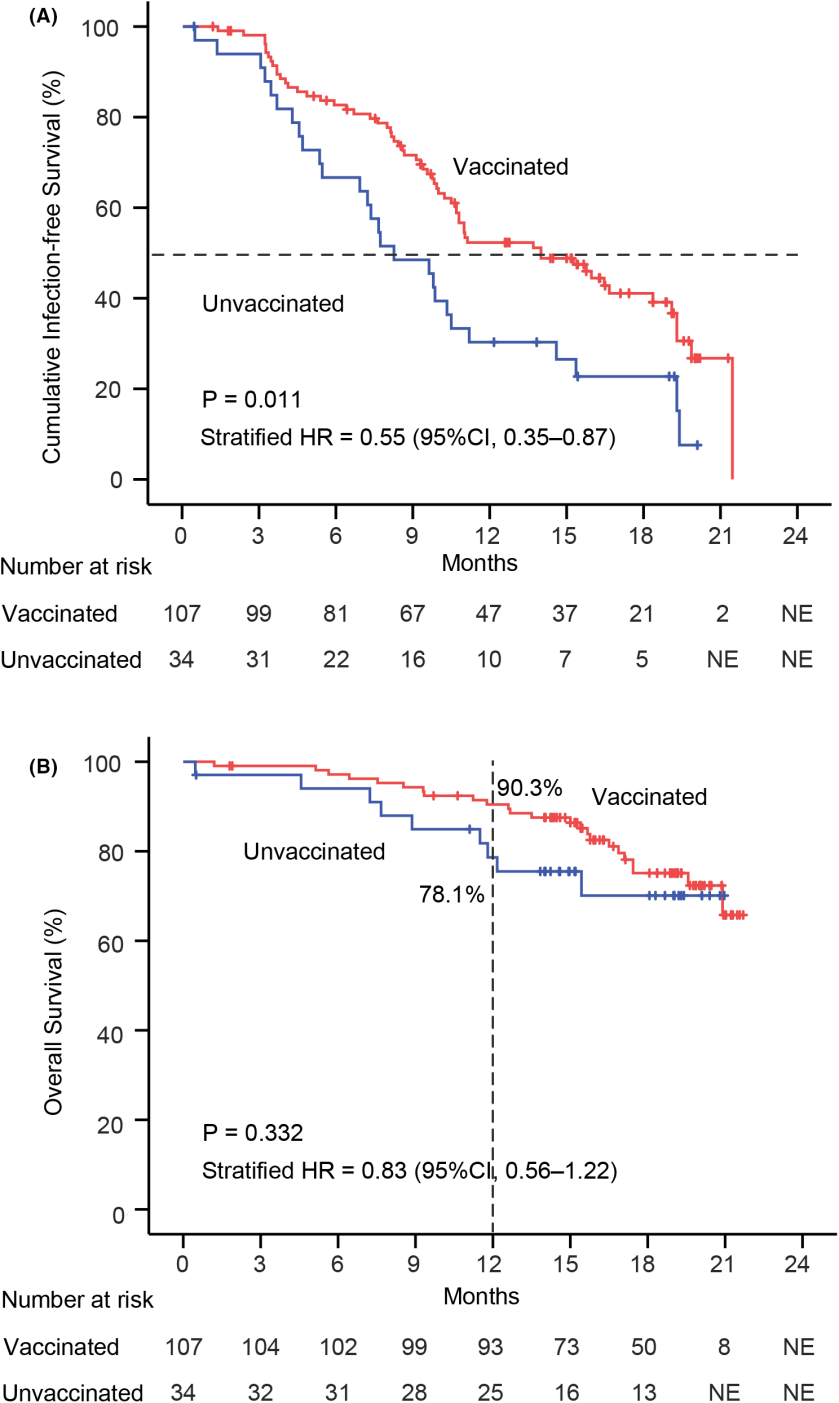
SARS‐CoV‐2 infection‐free survival and overall survival in patients with hepatocellular carcinoma. (A) SARS‐CoV‐2 infection‐free survival; (B) Overall survival.

Median infection‐free survival in the vaccinated group (14.0 months, 95% CI: 9.3–18.7 months) was significantly longer than that in the unvaccinated group (8.3 months, 95% CI: 5.5–11.0 months) (*p* = 0.010) (Figure 1A). In addition, the SARS‐CoV‐2 infection rate in the vaccinated group (*n* = 61, 57.0%) was significantly lower than that in the unvaccinated group (*n* = 27, 79.4%, *p* = 0.033). The number of patients with severe or very severe SARS‐CoV‐2 infection in the vaccinated group (*n* = 4, 3.7%) was relatively lower than that in the unvaccinated group (*n* = 4, 11.8%, *p* = 0.096). The univariate and multivariate COX regression analyses revealed that COVID‐19 vaccination (hazard ratio (HR) = 0.47, 95% CI: 0.29–0.77, *p* = 0.002), ECOG performance status = 0 (HR = 2.06, 95% CI: 1.05–4.04, *p* = 0.035), and extrahepatic spread (HR = 0.28, 95% CI: 0.09–0.95, *p* = 0.041) were the independent predictive factors for infection‐free survival (Table 1).

**TABLE 1:** Univariate and multivariate analyses for SARS‐CoV‐2 infection‐free survival.

	Univariate		Multivariate	
	HR (95% CI)	*p*‐Value	HR (95% CI)	*p*‐Value
Age (≤60 vs. >60)	1.16 (0.76, 1.78)	0.487		
Sex (male vs. female)	0.96 (0.56, 1.63)	0.881		
Occupation (managers or professionals vs. others)	0.73 (0.47, 1.16)	0.183	0.71 (0.44, 1.16)	0.175
Ethnic group (Han vs. others)	1.24 (0.39, 3.93)	0.717		
Household size (≤3 vs. >3)	0.76 (0.50, 1.17)	0.211		
Public transport use (yes vs. no)	1.22 (0.50, 3.02)	0.664		
HBV infection (yes vs. no)	1.14 (0.72, 1.79)	0.585		
Child‐Pugh class (A vs. B)	1.24 (0.64, 2.41)	0.517		
ECOG performance status (0 vs. ≥1)	1.97 (1.07, 3.64)	0.029	2.06 (1.05, 4.04)	0.035
BCLC stage (0/A vs. B/C/D)	1.35 (0.88, 2.06)	0.165	0.76 (0.45, 1.29)	0.312
Ascites (yes vs. no)	0.56 (0.28, 1.12)	0.099	0.73 (0.35, 1.53)	0.404
Maximum tumor diameter (≤5 vs. >5 cm)	1.30 (0.84, 2.01)	0.235	1.10 (0.66, 1.82)	0.724
Number of lesions (≤2 vs. >2)	1.16 (0.73, 1.82)	0.539		
Portal invasion (yes vs. no)	1.14 (0.62, 2.11)	0.676		
Extrahepatic spread (yes vs. no)	0.34 (0.11, 1.08)	0.068	0.28 (0.09, 0.95)	0.041
Cirrhosis (yes vs. no)	0.98 (0.64, 1.49)	0.924		
Hypertension (yes vs. no)	0.65 (0.40, 1.08)	0.096	0.62 (0.37, 1.03)	0.068
Diabetes (yes vs. no)	0.80 (0.50, 1.31)	0.804		
AFP (≤400 ng/mL vs. >400 ng/mL)	0.87 (0.54, 1.40)	0.564		
Immunotherapy (yes vs. no)	1.01 (0.55, 1.88)	0.956		
COVID‐19 vaccination (yes vs. no)^a^	0.55 (0.35, 0.87)	0.011	0.47 (0.29, 0.77)	0.002

*Note*: Candidate variables with a *p* < 0.20 on univariate analysis were included in multivariable analysis.

Abbreviations: AFP, alpha‐fetoprotein; BCLC, Barcelona Clinic Liver Cancer staging system; ECOG, European Cooperative Oncology Group; HBV, hepatitis B virus; HR, hazard ratio.

^a^ COVID‐19 vaccination represents the number of patients who received at least one dose of COVID‐19 vaccine.

### 3.5 Overall survival and COVID‐19 vaccination

Twenty‐four (22.4%) patients in the vaccinated group and nine (26.5%) patients in the unvaccinated group died during the follow‐up period (*p* = 0.719). The causes of mortality in the vaccinated group were tumor progression (*n* = 11), complications of cirrhosis (*n* = 12), and treatment‐related complications (*n* = 1). The causes of mortality in the unvaccinated group were tumor progression (*n* = 5), complications of cirrhosis (*n* = 3), and SARS‐CoV‐2 infection (*n* = 1). The overall survival at 12 months was 90.3% in the vaccinated group and 78.1% in the unvaccinated group (*p* = 0.070). The median overall survival was not reached in the two groups. The log‐rank analysis of the overall survival showed no significant difference between the two groups according to the data (*p* = 0.332) (Figure 1B). The univariate and multivariate COX regression analyses revealed that maximum tumor diameter ≤5 cm (HR = 0.25, 95% CI: 0.10–0.64, *p* = 0.004), portal invasion (HR = 2.51, 95% CI: 1.02–6.16) and SARS‐CoV‐2 infection (HR = 3.12, 95% CI: 1.25–7.84, *p* = 0.015) were the independent predictive factors for overall survival (Table S3).

## 4 DISCUSSION

Previous studies have shown that the safety and immunogenicity of the COVID‐19 vaccine are acceptable in patients with HCC.^10^, ^11^ However, the efficacy and prognostic factors of COVID‐19 vaccines in patients with HCC remain unclear. In the current study, we found that in patients with HCC, (1) COVID‐19 vaccines could effectively decrease the occurrence of SARS‐CoV‐2 infection; (2) COVID‐19 vaccination, ECOG performance status ≥1, and extrahepatic spread were the independent protective factors for the SARS‐CoV‐2 infection; and (3) SARS‐CoV‐2 infection, portal invasion, and maximum tumor diameter >5 cm were the independent risk factors for overall survival. There is a 53% lower risk of SARS‐CoV‐2 infection in patients with COVID‐19 vaccination, a 72% lower risk of SARS‐CoV‐2 infection in patients with extrahepatic spread, and a 2.06 times higher risk of SARS‐CoV‐2 infection in patients with ECOG performance status = 0. We suspected that the inability and restrictions in social contacts could be the major reasons for the lower infection rate in HCC patients with poor performance status. Nevertheless, this hypothesis requires further substantiation. There is a 68% lower risk of mortality in patients with SARS‐CoV‐2 infection, and 75% lower risk of mortality in patients with a maximum tumor diameter ≤5 cm, and a 2.51 times higher risk of death in patients with portal invasion. In addition, the occurrence of severe SARS‐CoV‐2 infection was nearly three times less common in vaccinated patients, which suggests that the COVID‐19 vaccines may also help to reduce the severity of SARS‐CoV‐2 infection. Similarly, several studies conducted in India have demonstrated that the COVID‐19 vaccines can modestly reduce the SARS‐CoV‐2 infection, and significantly decrease the severe SARS‐CoV‐2 infection.^12^, ^13^, ^14^ There are some limitations to this study. First, the sample size is relatively small (*n* = 141). Second, the majority of patients (99.3%) in the current study received inactivated COVID‐19 vaccines. Consequently, further studies focusing on the efficacy of the COVID‐19 mRNA or viral vectored vaccines are warranted in the future. Third, patients with prior SARS‐CoV‐2 infection were not included in this study. In this population, Kaur et al. conducted several excellent studies and found that the COVID‐19 vaccines could modestly reduce the SARS‐CoV‐2 infection.^13^, ^15^, ^16^ Fourthly, the vaccine‐related adverse reactions were not classified into short‐term or long‐term adverse reactions. Kaur et al. found a higher risk of long‐term adverse events after receiving COVID‐19 vaccines in patients with a history of SARS‐CoV‐2 infection.^15^, ^16^ Therefore, further studies focusing on patients with prior SARS‐CoV‐2 infection and the long‐term adverse reactions are warranted. At last, as the median overall survival was not reached, the findings about overall survival should be interpreted with caution. In conclusion, the COVID‐19 vaccines could effectively reduce the SARS‐CoV‐2 infection in patients with HCC.

## AUTHOR CONTRIBUTIONS

**He Zhao:** Conceptualization (equal); funding acquisition (equal); writing – original draft (lead); writing – review and editing (equal). **Ying Li:** Conceptualization (equal); formal analysis (equal); writing – review and editing (equal). **Pengfei Tian:** Data curation (equal); formal analysis (equal). **Wei Sun:** Data curation (equal); software (equal). **Yingen Luo:** Data curation (equal); formal analysis (supporting). **Xiaowu Zhang:** Data curation (supporting); formal analysis (equal). **Jingui Li:** Data curation (equal); formal analysis (supporting). **Tao Gong:** Data curation (equal); formal analysis (equal). **Zhengqiang Yang:** Data curation (equal); formal analysis (supporting). **Peng Song:** Data curation (supporting); formal analysis (supporting). **Xiao Li:** Conceptualization (equal); funding acquisition (lead); methodology (equal); project administration (equal); writing – review and editing (equal).

## FUNDING INFORMATION

This work was supported by the National Natural Science Fund of China [Grant number: 82330061 to X.L.], the CAMS Initiative for Innovative Medicine [Grant number: 2021‐I2M‐1‐015‐3 to X.L.], the National Health Commission Capacity Building And Continuing Education Center Annual Project [Grant number: GWJJ2024100302 to H.Z.] and the Beijing Hope Run Special Fund of Cancer Foundation of China [Grant number: LC2021C03 to Y.L].

## CONFLICT OF INTEREST STATEMENT

All of the authors have no conflict of interests.

## ETHICS STATEMENT

The study was approved by the local ethics committee of Cancer Hospital, Chinese Academy of Medical Sciences, and registered in the Chinese Clinical Trial Registry (identifier: ChiCTR2200056326).

## Supporting information


Figure S1.

Table S1.

## Data Availability

The datasets used and analyzed during the current study are available from the corresponding author on reasonable request.
