# Supplementary figures and images for: Efficacy and prognostic factors of COVID‐19 vaccine in patients with hepatocellular carcinoma: Analysis of data from a prospective cohort study
Source: Cancer Med. 2024 Aug 9;13(15):e70068. doi: 10.1002/cam4.70068 (PMC11310663; doi:10.1002/cam4.70068)

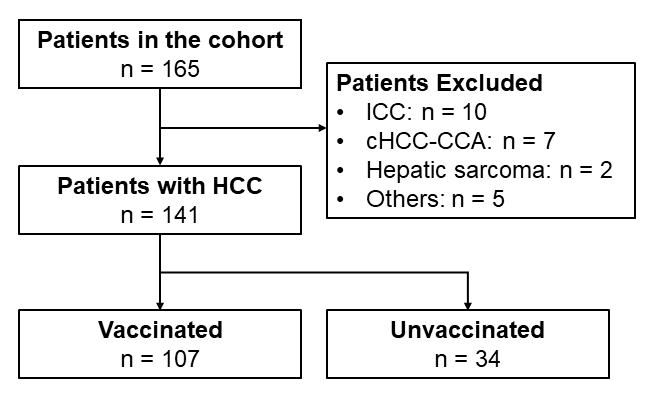

Supplement: Supplementary file 1 — Figure S1. [file CAM4-13-e70068-s001.tif]
