# Supplementary material for: Efficacy and prognostic factors of COVID‐19 vaccine in patients with hepatocellular carcinoma: Analysis of data from a prospective cohort study
Source: Cancer Med. 2024 Aug 9;13(15):e70068. doi: 10.1002/cam4.70068 (PMC11310663; doi:10.1002/cam4.70068)
Supplement: Supplementary file 2 — Table S1. [file CAM4-13-e70068-s002.docx]

| **Supplementary Table 1.** Baseline Characteristics | | | | |
| --- | --- | --- | --- | --- |
|  | Vaccinated  n = 107 | Unvaccinated  n = 34 | P Value |  |
| Age (years) | 59.2 ± 11.0 | 62.1 ± 11.5 | 0.193 |  |
| Male Sex | 85 (79.4) | 28 (82.4) | 0.711 |  |
| Occupation† |  |  | 0.301 |  |
| Managers or professionals | 32 (29.9) | 15 (44.1) |  |  |
| Workers or operators | 53 (49.5) | 14 (41.2) |  |  |
| Elementary occupations or others | 22 (20.6) | 5 (14.7) |  |  |
| Ethnic group‡ |  |  | 0.754 |  |
| Han | 103 (96.3) | 33 (97.1) |  |  |
| Manchu | 2 (1.9) | 0 (0) |  |  |
| Mongolian | 1 (0.9) | 1 (2.9) |  |  |
| Tibetan | 1 (0.9) | 0 (0) |  |  |
| Household size | 3.6 ± 0.9 | 3.8 ± 1.2 | 0.252 |  |
| Public transport use | 100 (93.5) | 32 (94.1) | > 0.999 |  |
| Etiology |  |  | 0.467 |  |
| HBV alone | 66 (61.7) | 17 (50.0) |  |  |
| Alcohol | 13 (12.1) | 6 (17.6) |  |  |
| HBV + Alcohol | 12 (11.2) | 4 (11.8) |  |  |
| HCV | 9 (8.4) | 2 (5.9) |  |  |
| Others | 7 (6.5) | 5 (14.7) |  |  |
| Child-Pugh Class |  |  | > 0.999 |  |
| A | 95 (88.8) | 12 (88.2) |  |  |
| B | 12 (11.2) | 4 (11.8) |  |  |
| ECOG Performance Status |  |  | 0.756 |  |
| 0 | 85 (79.4) | 26 (76.5) |  |  |
| 1 | 16 (15.0) | 5 (14.7) |  |  |
| 2 | 5 (4.7) | 2 (5.9) |  |  |
| 3 | 1 (0.9) | 1 (2.9) |  |  |
| BCLC Stage |  |  | 0.044 |  |
| 0 | 6 (5.6) | 5 (14.7) |  |  |
| A | 34 (31.8) | 9 (26.5) |  |  |
| B | 33 (30.8) | 4 (11.8) |  |  |
| C | 33 (30.8) | 15 (44.1) |  |  |
| D | 1 (0.9) | 1 (2.9) |  |  |
| Ascites | 13 (12.1) | 6 (17.6) | 0.596 |  |
| Maximum Tumor Diameter (cm) | 5.6 ± 5.0 | 5.5 ± 5.4 | 0.954 |  |
| Number of Lesions | 2 (1,3) | 2 (1,3) | 0.913 |  |
| Portal Invasion | 18 (16.8) | 8 (23.5) | 0.380 |  |
| Extrahepatic Spread | 8 (7.5) | 4 (11.8) | 0.669 |  |
| Cirrhosis | 54 (50.5) | 14 (41.2) | 0.345 |  |
| Hypertension | 36 (33.6) | 7 (20.6) | 0.150 |  |
| Diabetes | 29 (27.1) | 10 (29.4) | 0.793 |  |
| Serum Albumin (g/L) | 38.8 ± 4.6 | 38.0 ± 4.6 | 0.354 |  |
| Total Bilirubin (μmol/L) | 18.5 (13.4, 23.9) | 21.5 (14.3, 27.7) | 0.167 |  |
| Prothrombin Time (seconds) | 12.1 ± .5 | 12.4 ± 1.4 | 0.285 |  |
| Serum Creatinine (μmol/L) | 69.3 ± 16.3 | 72.2 ± 19.6 | 0.383 |  |
| AFP (ng/mL) | 6244 ± 34915 | 14297 ± 36116 | 0.250 |  |
| Median follow-up (days) | 488 ± 133 | 433 ± 164 | 0.075 |  |
| †Occupations were classified according to the International Classification of Occupations (version 8). ‡ All patients were Asian. HBV = hepatitis B virus; HCV = hepatitis C virus; ECOG = European Cooperative Oncology Group; BCLC = The Barcelona Clinic Liver Cancer staging system; AFP = Alpha-fetoprotein. | | | | |

| **Supplementary Table 2.** Characteristics of COVID-19 Vaccination | |  |
| --- | --- | --- |
| Items | n = 107 | |
| Number of All Doses | 259 | |
| COVID-19 Vaccination Status |  | |
| One Dose | 7 (6.5) | |
| Two Doses | 48 (44.9) | |
| Booster Vaccination | 52 (48.6) | |
| Type of Vaccine |  | |
| CoronaVac (Sinovac Life Sciences, China) | 52 (48.6) | |
| BBIBP-CorV (Beijing Institute of Biological Products, China) | 40 (37.4) | |
| Zifivax (Anhui Zhifei Longcom, China) | 7 (6.5) | |
| Others (Convidecia, BNT162b2, et al) | 8 (7.5) | |
| Vaccine-related Adverse Reactions (% all doses) |  | |
| Grade 1-2 | 35 (13.5) | |
| Grade 3 | 2 (0.8) | |
| Type of the Vaccine-related Adverse Reactions (% all doses) |  | |
| Local Pain Around the Injection Site | 22 (8.5) | |
| Fatigue | 6 (2.3) | |
| Vertigo | 4 (1.5) | |
| Cough | 3 (1.2) | |
| Sleepiness | 2 (0.8) | |
| Fever | 2 (0.8) | |
| Psoriasis Aggravation | 1 (0.4) | |

| **Supplementary Table 3.** Univariate and Multivariate Analyses for Overall Survival | | | | | |  |
| --- | --- | --- | --- | --- | --- | --- |
|  | Univariate |  |  | Multivariate |  | |
|  | HR (95%CI) | P value |  | HR (95%CI) | P value | |
| Age (≤60 vs. >60) | 1.29 (0.65, 2.59) | 0.470 |  |  |  | |
| Sex (male vs. female) | 0.46 (0.16, 1.32) | 0.151 |  | 0.70 (0.22, 2.21) | 0.549 | |
| Occupation (managers or professionals vs. others) | 0.78 (0.37, 1.65) | 0.522 |  |  |  | |
| Ethnic group (Han vs. others) | 21.8 (0.02, 22798.65) | 0.384 |  |  |  | |
| Household size (≤3 vs. >3) | 0.73 (0.37, 1.45) | 0.373 |  |  |  | |
| Public transport use (yes vs. no) | 0.72 (0.22, 2.35) | 0.581 |  |  |  | |
| HBV Infection (yes vs. no) | 1.94 (0.84, 4.51) | 0.121 |  | 1.82 (0.74, 4.52) | 0.195 | |
| Child-Pugh Class (A vs. B) | 3.26 (1.47, 7.28) | 0.004 |  | 1.42 (0.44, 4.66) | 0.559 | |
| ECOG Performance Status (0 vs. ≥1) | 0.48 (0.23, 0.99) | 0.048 |  | 1.32 (0.52, 3.40) | 0.562 | |
| BCLC Stage (0/A vs. B/C/D) | 0.27 (0.11, 0.67) | 0.004 |  | 1.01 (0.31, 3.33) | 0.989 | |
| Ascites (yes vs. no) | 1.75 (0.76, 4.04) | 0.189 |  | 0.43 (0.15, 1.23) | 0.114 | |
| Maximum Tumor Diameter (≤5 vs. >5cm) | 0.19 (0.08, 0.43) | <0.001 |  | 0.25 (0.10, 0.64) | **0.004** | |
| Number of Lesions (≤2 vs. >2) | 0.50 (0.25, 0.99) | 0.049 |  | 0.85 (0.38, 1.89) | 0.692 | |
| Portal Invasion (yes vs. no) | 4.09 (1.99, 8.42) | <0.001 |  | 2.51 (1.02, 6.16) | **0.044** | |
| Extrahepatic Spread (yes vs. no) | 4.78 (2.14, 10.66) | <0.001 |  | 2.30 (0.68, 7.83) | 0.182 | |
| Cirrhosis (yes vs. no) | 1.03 (0.52, 2.06) | 0.927 |  |  |  | |
| Hypertension (yes vs. no) | 1.53 (0.76, 3.08) | 0.235 |  |  |  | |
| Diabetes (yes vs. no) | 1.35 (0.66, 2.79) | 0.415 |  |  |  | |
| AFP (≤400ng/mL vs. >400ng/mL) | 2.10 (1.05, 4.16) | 0.035 |  | 1.05 (0.47, 2.34) | 0.910 | |
| Immunotherapy (yes vs. no) | 1.21 (0.43, 3.45) | 0.719 |  |  |  | |
| COVID-19 Vaccination (yes vs. no) | 0.69 (0.32, 1.48) | 0.336 |  |  |  | |
| SARS-CoV-2 Infection (yes vs. no) | 3.69 (1.80, 7.55) | <0.001 |  | 3.12 (1.25, 7.84) | **0.015** | |
| Candidate variables with a p value <0.20 on univariate analysis were included in multivariable analysis. HR = hazard ratio; HBV = hepatitis B virus; ECOG = European Cooperative Oncology Group; BCLC = Barcelona Clinic Liver Cancer staging system; AFP = Alpha-fetoprotein. | | | | | |  |
